# On Gram-Positive- and Gram-Negative-Bacteria-Associated Canine and Feline Skin Infections: A 4-Year Retrospective Study of the University Veterinary Microbiology Diagnostic Laboratory of Naples, Italy

**DOI:** 10.3390/ani11061603

**Published:** 2021-05-29

**Authors:** Francesca Paola Nocera, Monica Ambrosio, Filomena Fiorito, Laura Cortese, Luisa De Martino

**Affiliations:** Department of Veterinary Medicine and Animal Production, University of Naples “Federico II”, Via F. Delpino 1, 80137 Naples, Italy; francescapaola.nocera@unina.it (F.P.N.); monica.ambrosio@unina.it (M.A.); filomena.fiorito@unina.it (F.F.)

**Keywords:** Gram-positive and Gram-negative bacteria, pet animals, skin infections, antibiotic resistance

## Abstract

**Simple Summary:**

Pet animals’ bacterial skin infections represent the main reason for presentation in small animal practice and are generally secondary complications of other pathological conditions. Pyoderma and otitis externa are generally caused by *Staphylococcus* spp., and particularly *Staphylococcus pseudintermedius* is often isolated from dogs and cats suffering from skin disorders. However, also Gram-negative bacteria, such as *Pseudomonas aeruginosa* and *Escherichia coli*, can be responsible for both otitis externa and pyoderma. Since multi-drug-resistant bacterial strains have become a relevant threat in veterinary medicine, this study aimed to identify the bacteria most frequently associated with the most common clinical cases of skin infections in dogs and cats attending the University Veterinary Teaching Hospital of Naples in the period from January 2016 to December 2019. Moreover, their antibiotic resistance profiles were evaluated, highlighting an increasing spread of multi-drug-resistant strains. It is worth noting that this spread may also concern humans because of their close contact with pets. Thus, it not only is of veterinary significance but also has zoonotic importance, with pets acting as reservoirs for humans, especially pet owners and veterinarians.

**Abstract:**

A 4-year retrospective study (2016–2019) of selected routine bacteriological examinations of the veterinary microbiology laboratory of the University Veterinary Teaching Hospital of Naples (Italy) was carried out. A total of 189 bacteriological samples were collected from 171 dogs and 18 cats suffering from skin infections. In dogs, the most common cutaneous infection was otitis externa, while pyoderma was found to be prevalent in cats. The number of recorded Gram-positive strains over the study period did not vary considerably from year to year and was always significantly higher (*p*-value = 0.0007) in comparison with Gram-negative bacterial isolations. In dogs, *Staphylococcus pseudintermedius* was the most common identified Gram-positive bacterium (65%), while *Pseudomonas aeruginosa* (36%) was the one among the isolated Gram-negative bacteria. In cats, coagulase-negative staphylococci were the most predominant isolated bacteria (47%). The phenotypic profiles of antibiotic resistance showed that most of the strains were resistant to amoxicillin–clavulanate, penicillin, clindamycin, and trimethoprim–sulfamethoxazole. Several multi-drug-resistant strains (35%) were detected in canine isolates. An updating of antibiotic resistance profiles of the main Gram-positive and Gram-negative bacteria principally associated with skin infections of pet animals is necessary to improve stewardship programs of veterinary hospitals and clinics.

## 1. Introduction

Dogs and cats are exposed to bacteria daily, and most of the time, their immune systems are able to fight them off without showing any sign of disease. Bacterial disease occurs when a pet’s immune system is weakened, and bacteria can replicate and spread in the pet’s body. Furthermore, cleanliness practices of pet owners are essential to prevent the onset of diseases. Pet animals’ skin infections represent the main reason for presentation in small animal practice and are generally secondary complications of other pathological conditions, such as allergies, atopic dermatitis, and adverse food reactions. When skin barrier dysfunction occurs, a propensity toward secondary bacterial infections is established [1,2].

Usually, most of Gram-positive or Gram-negative infections are caused by the normal resident microflora of the skin, mucous membranes, and gastrointestinal tract. Cases of otitis, respiratory, urinary, and dermatological infections in pets involve principally both groups of bacteria. Pyoderma and otitis externa can affect dogs of any age or gender and are usually caused by *Staphylococcus* spp. [3,4]. Other bacteria commonly associated with otitis include *Pseudomonas aeruginosa*, *Escherichia* *coli* (*E. coli*), *Streptococcus* spp., *Proteus mirabilis*, *Enterococcus* spp., and *Corynebacterium* spp. [5]. Some bacteria, such as *Staphylococcus* spp. and *Pseudomonas* spp., may produce biofilm, which may increase the ability of these pathogens to resist antibiotics [6]. Particularly, the opportunistic Gram-positive bacterium, skin commensal or pathogen, *Staphylococcus pseudintermedius* (*S. pseudintermedius*) is considered the main causative agent of skin and ear infections in small animals [7,8,9]. Even though Gram-negative bacteria, such as *E. coli* and *Pseudomonas aeruginosa*, are prevalent as bacterial agents of canine and feline urinary tract infections [10], they are increasingly reported to be responsible for secondary skin infections, not only in humans [11], but also in small animals [12,13]. Study data on the prevalence and antibiotic resistance profiles of Gram-positive and Gram-negative bacteria isolated from owned dogs and cats referred daily to the teaching hospital appear to be not numerous in Italy. Antibiotics, important tools for the therapy of infectious bacterial diseases in companion animals, are typically used to treat infections, but antibiotic susceptibility testing is rarely requested. An Italian study conducted 10 years ago highlighted that in a veterinary teaching hospital, less than 5% of antibiotic prescriptions were made following antibiotic sensitivity test results [14]. In fact, antimicrobial therapy appears to be mainly empirical rather than based on antimicrobial susceptibility testing. This test should always be performed, especially in cases of recurrent infections, such as skin or urinary infections, where the rearing of resistant microorganisms is favored. Indeed, it has been reported that antibiotic-resistant bacterial infections commonly affect the skin, the gastrointestinal tract, the urinary tract, or the respiratory tract [15]. Furthermore, in our previous study, performed at the University Veterinary Teaching Hospital, antimicrobial prescription data were collected before and after the mandatory use of veterinary electronic prescription, highlighting that broad-spectrum antimicrobials such as amoxicillin–clavulanate and first-generation cephalosporins are the antibiotics more frequently administered in dogs and cats [16]. Thus, trends in antibiotic resistance among major bacterial pathogens isolated from pets are important to know, in order to recognize the bacterial infections that are minimally or no longer responsive to commonly used antibiotics. In other words, these bacteria, resistant to antibiotics, especially the ones with zoonotic potential, represent a worrisome threat to public health, since they could be transmitted to other pets or to humans [17].

Recently, a first study on the prevalence of bacterial pathogens and their antimicrobial resistance sampled from dogs and cats at the College of Veterinary Medicine Teaching Hospital of Colombia was published [18], and a similar study based on a large number of clinical cases collected within the Iberian Peninsula was published [5].

In recent years, in our hospital, request for bacteriological examinations before a therapy prescription has increased. Probably recommendations for prudent use of antimicrobials have allowed the increase of bacteriological tests. In addition, a more in-depth bacteriological diagnosis to know the molecular characterization, the main sequence type, the clonal spread of new variants or new approaches to conventional therapies was carried out [9,19]. It is more and more important to know the distribution of pathogens and their antibiotic resistance profile in pets, especially for their close contact with people, which offers favorable conditions for interspecies transmission of multi-drug-resistant bacteria [20]. Dogs are the major reservoirs for zoonotic infections that can be transmitted to humans by infected saliva, aerosols, contaminated urine or feces, and direct contact with the dog, and several case reports suggest household transmission of resistant strains between pets and their owners [21].

Here, clinical and laboratory findings and outcomes of animals with Gram-negative versus Gram-positive skin infections and susceptibility testing performed for the right choice of antibiotic treatment were compared. 

## 2. Materials and Methods

### 2.1. Informed Consent

This work did not involve the use of animals; instead, only noninvasive skin swab samples were obtained from animals being investigated for clinical reasons and for their benefit. Therefore, informed consent was not required.

### 2.2. Ethics Statement

No animal was used in the study. All the reports used had been collected for routine veterinary investigations. The design of this study was approved by the Ethical Animal Care and Use Committee of the University of Naples Federico II (certificate number PG/2021/0035101), in compliance with the Italian Legislative Decree 26/2014, Article 2.

### 2.3. Sample Collection

This research was conducted through the analysis of the database belonging to the Laboratory of Microbiology of the Department of Veterinary Medicine and Animal Production (University of Naples Federico II, Italy) from January 2016 to December 2019. All specimens were collected from clinically ill patients, precisely from independent cases of otitis or pyoderma, involving dogs and cats referred to the University Veterinary Teaching Hospital called Ospedale Veterinario Universitario Didattico (OVUD) of the above-mentioned department.

Samples were plated on blood agar base supplemented with 5% sheep blood, selective medium used for the isolation of Gram-positive microorganisms; on mannitol salt agar (MSA), selective medium to identify staphylococci; and on MacConkey agar (MCA), selective and differential medium to grow Gram-negative bacteria, which were incubated aerobically at 37 °C for 24–48 h. The plates were all microbiological media from Oxoid Ltd., Basingstoke, Hampshire, UK. Each morphotype colony was subjected to Gram staining, catalase, oxidase, and coagulase tests, followed by biochemical identification with the API system (BioMerieux, Marcy l’Etoile, France) according to the manufacturer’s instructions. The species identification by miniaturized biochemical tests was accepted when probability was >88%.

Pure colonies were stored into tryptone soy broth supplemented with 30% glycerol at −20 °C for future analysis.

The laboratory database refers to the exam files containing animal identification items (name, ID number, species, breed, sex, age, material collected, clinical diagnosis, and previous antibiotic intake), identification of the isolated bacteria, and results of the diameter of the bacterial growth inhibition zones obtained by Kirby–Bauer disk diffusion susceptibility tests. For Gram-positive and Gram-negative bacteria, the following antimicrobials were tested: amoxicillin–clavulanate (AMC, 20/10 µg), ampicillin (AMP, 10 μg), gentamicin (CN, 10 μg), imipenem (IMI, 10 μg), enrofloxacin (ENR, 5 μg), marbofloxacin (MAR, 5 μg), pradofloxacin (PRA, 5 μg), erythromycin (E, 15 μg), tetracycline (TE, 30 μg), trimethoprim–sulfamethoxazole (SXT, 25 μg). Gram-positive bacteria were also tested for penicillin (P, 10 IU) and clindamycin (CD, 2 μg). Moreover, staphylococci were tested for susceptibility to oxacillin (OX, 1 μg) as an indicator for methicillin resistance. The tested antibiotics belonged to 8 different classes. The interpretation of antimicrobial resistance/susceptibility was performed according to Clinical Laboratory Standards Institute guidelines [22] for clindamycin, enrofloxacin, erythromycin, gentamicin, marbofloxacin, oxacillin, penicillin, pradofloxacin, tetracycline, and trimethoprim–sulfamethoxazole, while for amoxicillin–clavulanate, ampicillin, and imipenem, the European Committee on Antimicrobial Susceptibility Testing guidelines were used [23]. 

Multidrug resistance was defined according to Magiorakos et al. [24] for bacteria showing resistance to at least 3 different antibiotic classes. 

The reference strains *S. pseudintermedius* ATCC 49444, *S. aureus* ATCC 33591, *E. coli* ATCC 25922, and *Pseudomonas aeruginosa* ATCC 15442 were used as quality controls.

All the information obtained from the house database was inserted into spreadsheets using Excel software, where it was separated by year of isolation, type of skin infection, bacterial species, and profiles of antimicrobial susceptibility. 

The bacteria were organized into two groups:

(I) Gram-positive bacteria: *Staphylococcus* spp., *Streptococcus* spp.;

(II) Gram-negative bacteria: *Pseudomonas* spp., *Enterobacterales*, and *Acinetobacter* spp.

### 2.4. Data Management

All diagnostic data generated by the bacteriology laboratory were recorded in a data-capturing format and entered into a Microsoft 365 Excel™ spreadsheet for subsequent analysis. Descriptive statistics were employed to analyze the proportions of each group (age, gender, skin infection types, Gram-positive and Gram-negative bacteria, antimicrobial drugs) related to dogs or cats. The graphics were made by Excel software also for the frequencies of antimicrobial resistance, which were determined using descriptive statistical analysis.

### 2.5. Statistical Analysis

The statistical significance level between bacteria groups was investigated using Fisher’s exact test (GraphPad Software Inc., Avenida De La Playa La Jolla, CA, USA). *p*-Values ≤ 0.05 were considered statistically significant at 95% confidence interval. 

## 3. Results

### 3.1. Gram-Positive- and Gram-Negative-Bacteria-Associated Pet Animal Skin Infections

**Figure 1:**
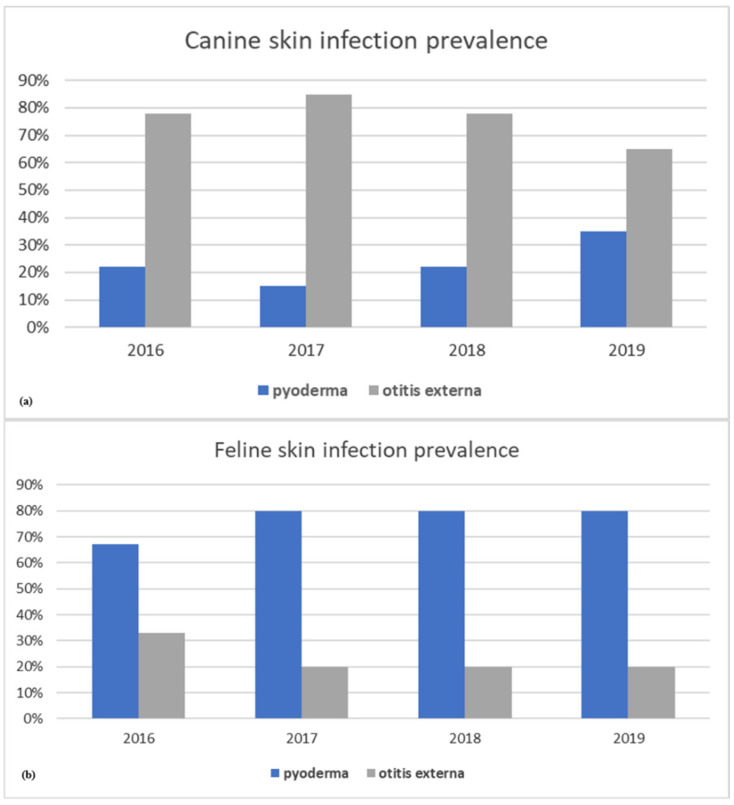
Prevalence of skin infection types by year in dogs (**a**) and cats (**b**).

In this 4-year retrospective study, a total 189 samples from cases of canine and feline skin infections were microbiologically analyzed. Year-wise distribution of pet animal skin infection type over the 4 years is shown in Figure 1a,b. In dogs, cases related to otitis externa were always extremely significantly (*p*-values < 0.001) higher compared with pyoderma ones (Figure 1a). Different from dogs, pyoderma samples in cats were the most frequently remitted to the lab (83%) and were particularly significantly (*p*-values < 0.001) higher than otitis externa ones (Figure 1b).

**Figure 2:**
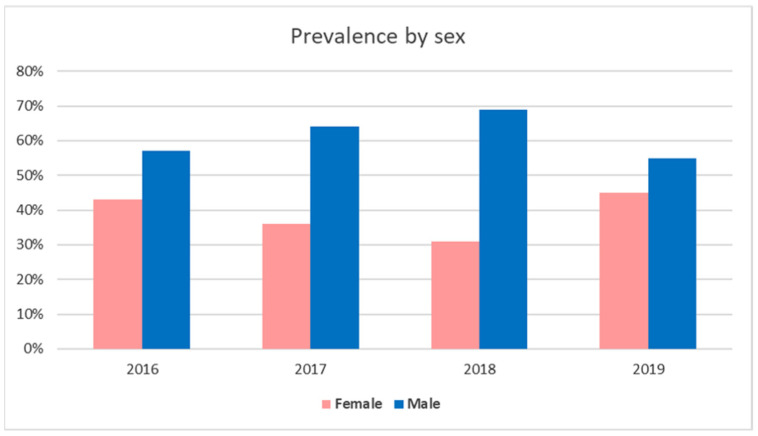
Prevalence of skin infection cases by study year associated with the sex of pets.

**Figure 3:**
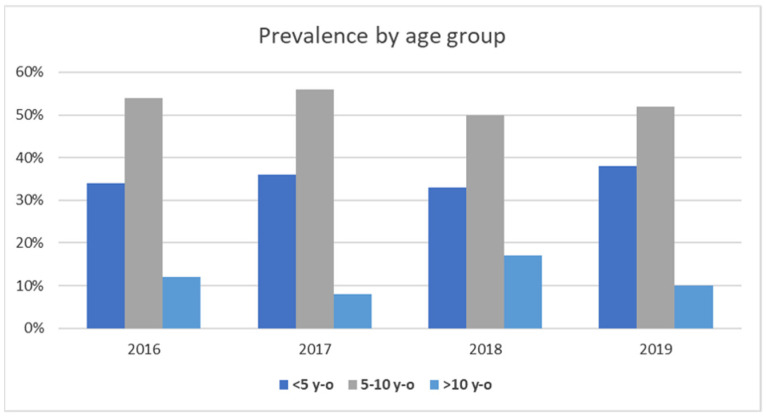
Prevalence of pet animal skin infections by age group.

The examinations involved 171 dogs (101 males and 70 females) and 18 cats (10 males and 8 females). The gender and age distributions in pet animals suffering from skin infections are reported in Figure 2 and Figure 3. Regarding gender, in the examined 4 years, skin infection frequency was always higher in males than females for both dogs and cats (Figure 2). Concerning age distribution, most of canine and feline patients were placed in the 5–10 age group, followed by the <5 age group (Figure 3).

The highest number of processed samples (73/189; 38.6%) was recorded in 2019, whereas the lowest number (35/189; 18.5%) was observed in 2016.

From all canine and feline processed samples, a total of 232 bacterial strains were isolated and classified in 148 Gram-positive bacteria (64%) and 84 Gram-negative bacteria (36%). Precisely, from a total of 213 canine bacterial isolates, 132 (62%) were Gram-positive strains, while 81 (38%) were Gram-negative strains. Among the 19 (8%) isolated feline strains, Gram-positive bacteria were the most prevalent isolates (16/19; 84%). Moreover, from 28/171 samples (16%) and 1/18 (6%) for dogs and cats, respectively, multiple bacteria were isolated. 

**Figure 4:**
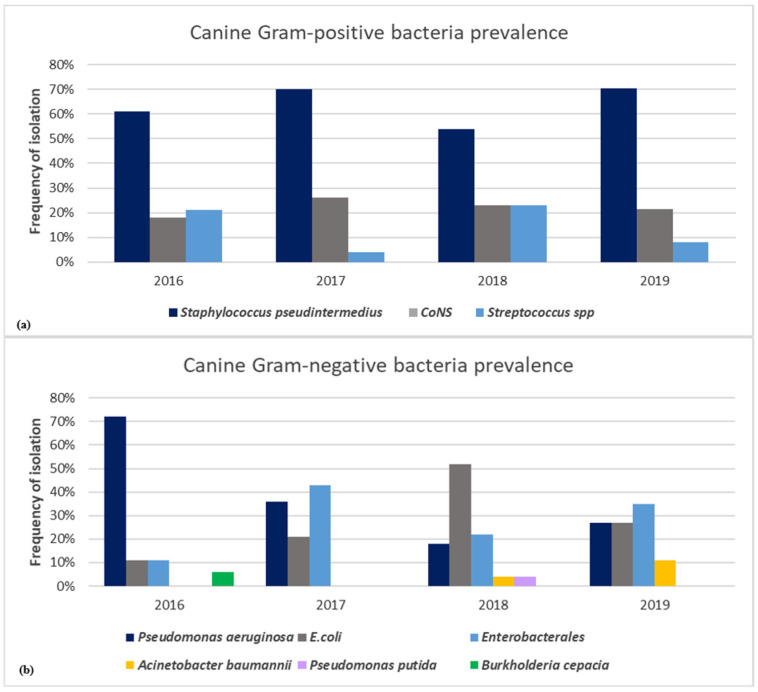
Frequency of isolation by year of Gram-positive (**a**) and Gram-negative (**b**) bacteria associated with canine skin infections.

In dogs, *S. pseudintermedius* was the most common identified Gram-positive bacterium (86/132; 65%), followed by *Pseudomonas aeruginosa* (29/81; 36%) for Gram-negative bacterium (Figure 4a,b). Among coagulase-negative *Staphylococcus* (CoNS) species (29/132; 22%), *S. xylosus*, *S. simulans*, *S. epidermidis*, *S. sciuri*, *S. chromogenes*, *S. hyicus*, and *S. cohnii* were identified, whereas among *Streptococcus* spp. (17/132; 13%), *Streptococcus canis*, *Streptococcus mitis*, *Streptococcus dysgalactiae*, and *Streptococcus agalactiae* were detected (Figure 4a). *E. coli* (24/81; 30%) and other *Enterobacterales* (22/81; 27%), such as *Klebsiella pneumoniae*, *Proteus mirabilis*, *Raoultella ornithinolytica*, *Enterobacter cloacae*, *Serratia marcescens*, and *Citrobacter youngae*, were the most predominant Gram-negative bacteria isolated in dogs (Figure 4b). 

We detected the presence of other pathogens belonging to the ESKAPE group, encompassing both Gram-positive and Gram-negative species, including *Enterococcus faecium*, *Staphylococcus aureus*, *Klebsiella pneumoniae*, *Acinetobacter baumannii*, *Pseudomonas aeruginosa*, and *Enterobacter* species. Particularly, *Acinetobacter baumannii* (*A. baumannii*) strains were isolated from dogs suffering from otitis externa in the years 2018 (4%) and 2019 (11%) (Figure 4b).

Different from dogs, over the 4 years of study, feline skin infections were caused principally by CoNS (9/19; 47%), *S. aureus* (4/19; 21%), and *S. pseudintermedius* (2/19; 11%), while a low frequency of 5.2% was recorded for *Streptococcus canis* (1/19), *Pseudomonas aeruginosa* (1/19), *E. coli* (1/19), and *Klebsiella pneumoniae* (1/19) strains.

**Figure 5:**
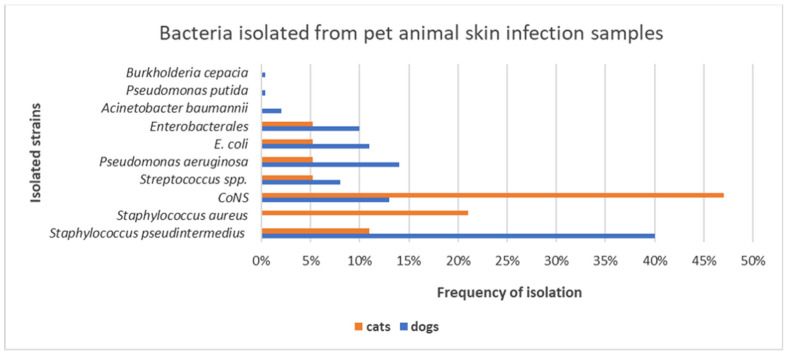
Frequency of isolation of bacterial species from skin infection samples in dogs and cats in the years 2016–2019.

The bacterial strains derived from otitis specimens were 170, of which 165/170 (97%) were of canine origin, and 5/170 (3%) of feline origin, while those derived from pyoderma samples were 62: 48/62 (77%) of canine origin and 14/62 (23%) of feline origin. From otitis and pyoderma specimens, infections by *Staphylococcus* spp. were highly detected in both dogs and cats. Precisely, the prevalence of CoNS was significantly higher in cats than in dogs, resulting in extreme statistical significance (p-value = 0.0032). However, dogs presented a larger bacterial diversity in skin infection samples than cats, as reported in Figure 5.

### 3.2. Gram-Positive and Gram-Negative Bacteria Antibiotic Resistance Profiles

In the present study, the antibiotic resistance profiles of canine *S. pseudintermedius*, CoNS, *Streptococcus* spp., *Pseudomonas aeruginosa*, *E. coli* and other *Enterobacterales*, and *Acinetobacter baumannii* strains were analyzed. The antibiotic resistance profiles of feline strains were not systemically analyzed due to the reduced number of the isolates collected from cats.

**Figure 6:**
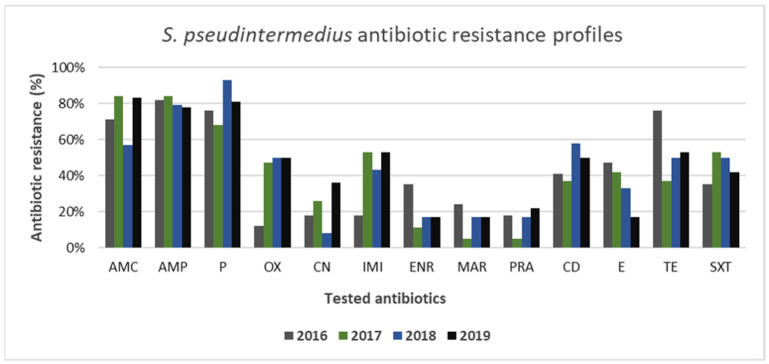
Antimicrobial resistance profiles of canine *S. pseudintermedius* strains to commonly used antibiotics.

Both canine Gram-positive and Gram-negative strains showed high resistance to tested antibiotics. Among the Gram-positive strains analyzed, the most resistant species was represented by *S. pseudintermedius*. As shown in Figure 6, the antimicrobial susceptibility profiles of *S. pseudintermedius* strains showed high resistance to amoxicillin–clavulanate, ampicillin, and penicillin being around 80% for all 4 years of study. Resistance to oxacillin was detected in 35 of 86 total isolated strains of *S. pseudintermedius* (41%). Precisely, *S. pseudintermedius* frequency of resistance to oxacillin ranged from 12% (2016) to 50% (2018–2019), displaying an alarming increase over the studied years. A worrying increasing resistance trend of this pathogen was observed also for gentamicin and imipenem, ranging from 18% to 36% and 18% to 53%, respectively. Resistance to erythromycin in *S. pseudintermedius* exhibited a relevant downward trend, ranging from 47% to 17%. A similar trend was observed also for enrofloxacin and tetracycline with values of resistance ranging from 35% to 17% and 76% to 53%, respectively. Clindamycin, marbofloxacin, pradofloxacin, and trimethoprim–sulfamethoxazole fluctuated lightly, ranging from 41% to 50%, 24% to 17%, 18% to 22%, and 35% to 42%, respectively. However, the overall prevalence of antimicrobial resistance among *S. pseudintermedius* isolates detected in this study appeared high, being few resistances with values below 20% (Figure 6). A multi-drug-resistance profile was present in 30% (26/86) of *S. pseudintermedius* isolates.

**Figure 7:**
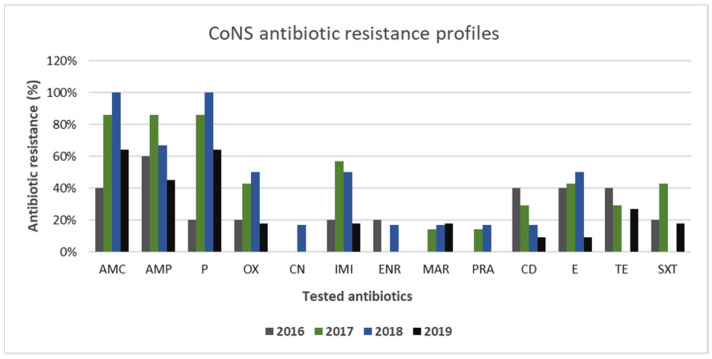
Antimicrobial resistance profiles of canine CoNS strains to commonly used antibiotics.

The antibiotic resistance profiles of CoNS strains isolated from canine samples are reported in Figure 7. The resistance frequencies for CoNS to amoxicillin–clavulanate and penicillin were higher than 80% in 2017–2018 with a value of 100% in 2018. Then, they were relevantly reduced for both antibiotics in 2019 (64%). CoNS resistance to oxacillin was lower than that for *S. pseudintermedius* strains, showing fluctuating values over the 4 years, but never higher than 50% (2018). Resistances to clindamycin, erythromycin, imipenem, tetracycline, and trimethoprim–sulfamethoxazole demonstrated a downward trend over the 4-year test period. The resistance rates of gentamicin, enrofloxacin, and marbofloxacin varied slightly over the studied period, with values always lower than 20%.

Multidrug resistance was found in 24% (7/29) of CoNS strains.

*Streptococcus* spp. strains presented the lowest levels of resistance in comparison with other Gram-positive bacteria. The highest antibiotic resistance values were recorded for amoxicillin–clavulanate, penicillin, and marbofloxacin in the years 2018–2019 (data not shown).

**Figure 8:**
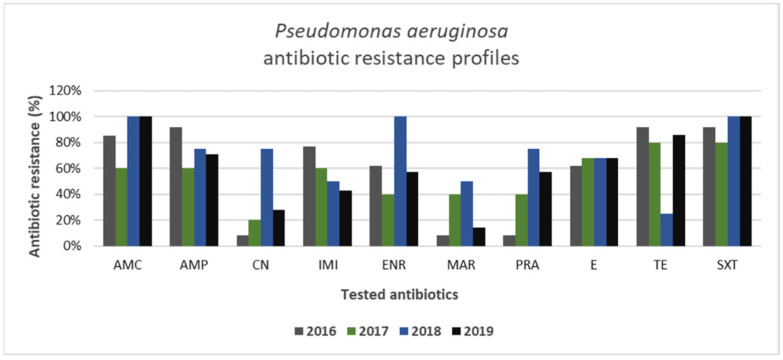
Antimicrobial resistance profiles of canine *Pseudomonas aeruginosa* strains to commonly used antibiotics.

Among Gram-negative bacteria isolated from canine specimens, *Pseudomonas aeruginosa* appeared to be the most resistant strain, presenting the highest levels of antibiotic resistance (Figure 8). Particularly, the highest values of resistance were recorded for amoxicillin–clavulanate and trimethoprim–sulfamethoxazole during the 4 years of study, reaching a resistance value of 100% for these antibiotics in the years 2018–2019. On the other hand, gentamicin, enrofloxacin, marbofloxacin, and pradofloxacin showed variable antibiotic resistance values over the years. Intriguingly, resistance to imipenem decreased from 77% to 43% during the years of study. Moreover, 79% of *Pseudomonas aeruginosa* isolates (23/29) were multi-drug-resistant strains.

**Figure 9:**
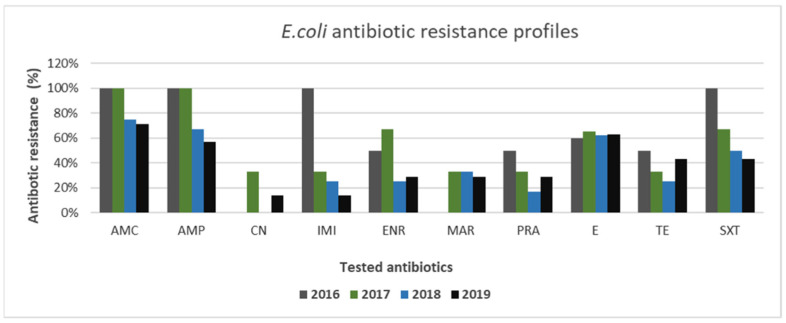
Antimicrobial resistance profiles of canine *E. coli* strains to commonly used antibiotics.

Within *Enterobacterales*, *E. coli* exhibited interesting profiles of antibiotic resistance. Particularly, as shown in Figure 9, resistance to imipenem in *E. coli* exhibited a downward trend, ranging from 100% to 14%. Similar decreasing trends were also observed for amoxicillin–clavulanate, ampicillin, enrofloxacin, and trimethoprim–sulfamethoxazole ranging from 100% to 71%, from 100% to 57%, from 50% to 29%, and from 100% to 43%, respectively. For marbofloxacin, pradofloxacin, and tetracycline, trends marginally changed during the considered years; meanwhile, the lowest levels of resistance were recorded for gentamicin. During 2016–2019, 46% (11/24) of multi-drug-resistant *E. coli* strains were identified.

**Figure 10:**
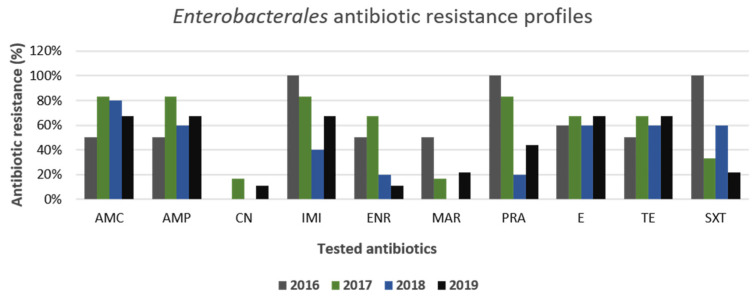
Antimicrobial resistance profiles of canine *Enterobacterales* strains to commonly used antibiotics.

Furthermore, the other Gram-negative bacteria isolated and belonging to the *Enterobacterales* group displayed antibiotic resistance profiles like *E. coli* (Figure 10). Precisely, the highest levels of resistance were observed for amoxicillin–clavulanate and ampicillin, while variable and decreasing trends were observed for antibiotics such as imipenem, trimethoprim–sulfamethoxazole, and the three fluoroquinolones, enrofloxacin, marbofloxacin, and pradofloxacin, during the 4-year test period. Similar to *E. coli*, for all *Enterobacterales* strains, the highest levels of susceptibility were obtained against gentamicin. In addition, multi-drug-resistance profiles were detected in 64% (14/22) of the strains.

## 4. Discussion

In pet animals, skin infections represent the main reason for presentation to veterinary examination, often leading to empirical antimicrobial prescription in small animal practices [14,25,26]. Thus, timely detection, identification, and antimicrobial susceptibility testing of causative pathogens and their surveillance are needed to improve the management of otitis externa and pyoderma in pets and to prescribe a successful therapy.

This study reports data on the prevalence of otitis externa and pyoderma in dogs and cats and the frequency of most isolated bacteria and evaluates their susceptibility profiles to the main antibiotics used in veterinary treatments in the period between January 2016 and December 2019. Moreover, this retrospective research can be a guideline for clinicians, especially those in our veterinary teaching hospital, in making rational decisions on the use of antibiotics, since empirical antimicrobial treatment is frequently given out to companion animals visiting hospital or veterinary health care facilities.

In this 4-year study period, a total of 189 auricular and cutaneous samples were examined, of which 171 and 18 were of canine and feline origin, respectively. However, a notable increase in the number of routine bacteriological examinations was observed in the 4th year of the study compared with the previous years, probably due to the information and conviction policy of our department on the need to request bacteriological analysis before prescribing antimicrobial therapies.

For the examined years, skin infections occurred predominantly in male patients aged from 5 to 10 years old in both dogs and cats, in accordance with other studies [27,28]. Precisely, in dogs most of the specimens were from ears (74%), while in cats they were from skin lesions (83%). Intriguingly, in cats we detected a higher number of pyoderma cases than in dogs, even though in the literature feline pyoderma is considered rare [29,30,31]. However, our results are in accordance with those reported by Yu et al. [28], which already highlighted an underestimation of this infection in cats.

*Staphylococcus* was the most isolated bacterial genus from skin infection samples of both canine and feline origins, confirming the role of *Staphylococcus* spp. as opportunistic pathogens of skin and mucous membrane sites. Since staphylococci are part of the natural skin microbiota [32], they are commonly reported as the leading cause of otitis externa, pyoderma, and postoperative wound infections in companion animals [33,34]. Moreover, according to the literature, also in this 4-year study, staphylococci, in particular CoNS (47%), *S. aureus* (21%), and *S. pseudintermedius* (11%), were found to be the main causative agents of feline skin infections [28,35,36,37].

*S. pseudintermedius* (40%), *Pseudomonas aeruginosa* (14%), CoNS (13%), *E. coli* (11%), *Enterobacterales* (10%), and *Streptococcus* spp. (8%) were the most frequently isolated microorganisms from the culture of canine specimens. Our findings are consistent with other reports, which define these bacteria as the most common isolates from dogs suffering from otitis externa and pyoderma [4,5,38,39,40,41,42,43,44]. Furthermore, the results of this study show a high prevalence (40%) of *S. pseudintermedius* among the other bacterial species, approving its role as the main causative agent of canine skin infections [4,45]. The high rate of colonization with *Staphylococcus* spp. in both dogs and cats may represent a public health issue, since the transmission of staphylococci from carrier or infected pet animals to humans has been documented [46,47,48].

*Pseudomonas aeruginosa* was the second most common isolated bacteria from canine samples. Our results agree with the study of Bourély et al. [44], which reported *S. pseudintermedius*, followed by *Pseudomonas aeruginosa*, as the most common isolated canine pathogens. By contrast, Budgen [40] described *Pseudomonas aeruginosa* as the most frequently isolated bacterium from canine otitis externa samples in Australia, followed by *S. pseudintermedius*, linking the higher prevalence of *Pseudomonas aeruginosa* to an increased diagnosis of chronic otitis externa.

The percentage of CoNS positivity (13%) among the processed samples highlights a relevant dog susceptibility to these bacterial species. Streptococci and *E. coli* and other *Enterobacterales*, such as *Proteus mirabilis*, *Klebsiella pneumoniae*, and *Enterobacter* spp., were also isolated from canine auricular and cutaneous samples. This finding is not surprising, as they are often involved in canine otitis externa and pyoderma [5]. Moreover, *A. baumannii* strains were isolated from dogs suffering from otitis externa in the years 2018 (4%) and 2019 (11%). This bacterial strain has become an important emerging pathogen in veterinary medicine, since it is more and more frequently associated with otitis, abscess, sepsis, urinary tract, and respiratory infections in pet animals [49,50,51]. In addition, *A. baumannii* zoonotic potential should not be underestimated with pet animals as potential reservoirs of *A. baumannii*, including those resistant to carbapenems [52,53].

Among the Gram-positive bacteria analyzed, the most resistant species was represented by *S. pseudintermedius*. High resistance to amoxicillin–clavulanate, ampicillin, and penicillin (>80%) has also been reported by other authors [45,47,54]. Regarding oxacillin resistance, it was detected in 41% (35/86) of the isolated strains, and the resistance level ranged from 12% (2016) to 50% (2018–2019), highlighting the increasing spread of methicillin-resistant *S. pseudintermedius* (MRSP) strains. Besides β-lactam antibiotics, multi-drug-resistance profiles were detected in 30% of *S. pseudintermedius* strains, which showed relevant resistance values also to other antibiotics approved in veterinary medicine and used for systemic treatment in dogs (gentamicin, imipenem, clindamycin, tetracycline, trimethoprim–sulfamethoxazole), confirming, thus, the multi-drug-resistance trend reported worldwide [54,55,56,57]. In this context, the emergence in dogs of MRSP, often associated with an even broader drug resistance, has become a great veterinary challenge [58] and has assumed new public health relevance due to its zoonotic potential. Particularly, veterinary environments (hospitals and clinics) seem to play an important role in the dissemination of MRSP between small animals and humans, especially people who have constant contact with pets [59,60].

CoNS spp., members of the normal flora of human and animal skin, have long been considered nonpathogenic, possessing fewer virulence properties than coagulase-positive *Staphylococcus* (CoPS) species. Recently, they have assumed an important role as pathogens in skin and soft tissue infections, overall, because of their increasing multi-drug-resistance profiles. In this study, CoNS displayed interesting antibiotic resistance profiles. CoNS showed high resistance to amoxicillin–clavulanate and penicillin, reaching 100% resistance in the years 2018–2019, while low resistance was shown for gentamicin, enrofloxacin, and marbofloxacin. However, 7/29 (24%) strains were found to be multi-drug-resistant strains. This result is of great interest, since CoNS spp. are known to be the reservoir of resistance genes; therefore, the resistances shown in this study could spread among pathogenic staphylococci, such as *S. pseudintermedius* and *S. aureus*, and increase difficulties in treating infections caused by multi-drug-resistant pathogens [61].

In this study, *Streptococcus* spp. strains presented the lowest levels of resistance in comparison with other Gram-positive bacteria. However, in the years 2018–2019 high levels of *Streptococcus* spp. resistance to amoxicillin–clavulanate, penicillin, and marbofloxacin were recorded, in contrast to other studies where high levels of *Streptococcus* spp. susceptibility also to these antibiotics were reported [5,34]. Furthermore, *Pseudomonas aeruginosa* showed the highest values of antibiotic resistance among Gram-negative bacteria, and 79% (23/29) of isolates were found to be multi-drug-resistant strains. *Pseudomonas* spp. are intrinsically resistant to many antibiotics, such as β-lactams, combinations with β-lactamase inhibitors, chloramphenicol, tetracycline, and trimethoprim–sulfamethoxazole [62], and are also known for their ability to rapidly acquire further resistances [63]. On the other hand, we observed that gentamicin, enrofloxacin, marbofloxacin, and pradofloxacin showed variable antibiotic resistance levels over the years. Precisely, *Pseudomonas aeruginosa* showed lower levels of resistance to gentamicin and marbofloxacin, but higher values of resistance to enrofloxacin, which is commonly used systemically in combination with a topical treatment to treat canine otitis caused by *Pseudomonas aeruginosa* [64]. The high level of resistance of *Pseudomonas aeruginosa* to enrofloxacin over the studied years is in accordance with results from many other studies [64,65,66]. These results suggest that gentamicin and marbofloxacin can be considered to have potential as antipseudomonal drugs [67].

In the present study, *E. coli* and the other *Enterobacterales* strains isolated from dogs showed similar antibiotic resistance profiles, with high levels of resistance to β-lactams. Interestingly, a decreasing trend of resistance was recorded for imipenem in the studied period, suggesting a reduced circulation of carbapenem-resistant *Enterobacteriaceae* in pet animals, even though these bacteria have been increasing rapidly worldwide [68,69] and are considered priority pathogens for which new antibiotics are urgently needed by the World Health Organization [70]. The lowest levels of resistance were observed for gentamicin, often used to topically treat otitis externa in small animals, and marbofloxacin. However, multidrug resistance was observed in 46% (11/24) and 64% (14/22) of *E. coli* and *Enterobacterales* strains, respectively.

Furthermore, it is worth noting that, as already reported by Kroemer et al. [71], in this study all the isolated strains showed good susceptibility to fluoroquinolones, mainly to marbofloxacin, which represents an effective antibiotic for the treatment of otitis, pyoderma, UTIs, and respiratory infections in pet animals. This is a reassuring result, considering that fluoroquinolones are considered critically important antibiotics in human medicine. The results of this study provide information on susceptibility profiles of bacterial isolates at the University Veterinary Microbiology Diagnostic Laboratory of Naples. Samples remitted to our microbiology laboratory may reflect clinical cases of dogs and cats within this city, and knowledge of the most frequently isolated bacteria from dog and cat infections and their associated antibiotic resistance profiles and trends is an important consideration to improve the appropriate use of antibiotics decreasing resistance selection pressure.

The findings of the present study highlight encouraging evidence that requests for bacteriological examinations increased in 2019, probably also due to the frequent organizations of seminars, workshops, and events on the importance of the antimicrobial resistance surveillance in our veterinary teaching hospital. In addition, in Italy since April 2019, veterinary electronic prescription has been mandatory for the prudent use of antibiotics. In fact, Article 3 of Law No. 167 of 20 November 2017 [72] provides the adoption in our country of a computerized system for the traceability of veterinary medicinal products and medicated feed. This new computerized system has replaced the paper form of prescription in the whole national territory, simplifying procedures, reducing administrative obligations, improving control activities, and re-elaborating data useful to contain antimicrobial resistance. All those favor the control of antimicrobial-resistant microorganisms isolated from pets. Although the true magnitude of antibiotic resistance in pets and other animals, as well as humans, is not fully known, pets can contribute to the spread of antibiotic resistance due to their close contact with humans and their status as family members in urban households. Antibiotic resistance is currently a public health problem, and it is mainly linked to inappropriate use of antibiotics in both humans and animals. Obviously, risk exists when the same antibiotic is used in both veterinary and human medicine or exhibits cross-resistance with other antibiotics used mainly in human medicine. Furthermore, great concern is presented toward zoonotic resistant bacteria, which can spread from animals to humans and vice versa, and have become a worrying threat in both veterinary and human medicine.

## 5. Conclusions

Request for antimicrobial susceptibility testing before antimicrobial prescription, which should always be performed, particularly before treatment of recurrent infections, has increased over the years. Mandatory use of electronic prescription can certainly help to control and combat antibiotic resistance, limiting both the improper use of antimicrobials and the spread of multi-drug-resistant pathogens [16]. Furthermore, the results of this work highlight the potential of pets as reservoirs of multi-drug-resistant pathogens that could spread to owners and veterinary personnel in veterinary hospitals and home environments. In conclusion, this type of study gives vision into the dynamics and the rate of bacteria isolations in pets, offering a stimulus for further investigation into the update of zoonotic bacteria associated with infectious diseases of small animals that, long since, fit within the old One Health concept. Moreover, this topic still deserves considerable monitoring to implement organized surveillance programs.

## Data Availability

Not applicable.
